# Pharmacological Insights into Cannabidiol for Wound Healing and Bone Regeneration

**DOI:** 10.1021/acsomega.5c13259

**Published:** 2026-04-13

**Authors:** Letícia P. L. Durão, Daniela M. Cunha, Marta M. A. Pereira, Erica D. de Avila, Patricia M. Maquera-Huacho

**Affiliations:** † Department of Diagnosis and Surgery, School of Dentistry, São Paulo State University (UNESP), 14801-903 Araraquara, Brazil; ‡ Department of Physiology and Pathology, School of Dentistry, São Paulo State University (UNESP), 14801-903 Araraquara, Brazil; § Department of Dental Materials and Prosthodontics, São Paulo State University (UNESP), 16015-050 Araçatuba, Brazil; ∥ Postgraduate Program in Dentistry, Federal University of Piaui, 64049-550 Teresina, Brazil; ⊥ Department of Diagnostic and Surgery, Araçatuba School of Dentistry, São Paulo State University (UNESP), 16015-050 Araçatuba, Brazil; # Department of Dental Materials and Prosthodontics, São Paulo State University (UNESP), 14801-903 Araraquara, Brazil; ∇ School of Dentistry, Universidad Católica de Santa María (UCSM), 04013 Arequipa, Peru

## Abstract

Cannabidiol (CBD), a major nonpsychoactive phytocannabinoid derived from *Cannabis sativa* L., has recently gained prominence for its broad pharmacological profile and emerging applications in regenerative medicine. Beyond its well-established neuroprotective, antiepileptic, anxiolytic, antipsychotic, anti-inflammatory, analgesic, and anticancer effects, CBD has demonstrated the capacity to modulate key biological processes involved in tissue repair. Increasing evidence indicates that CBD promotes wound healing by regulating inflammatory responses, cellular proliferation, and extracellular matrix remodeling through interactions with cannabinoid and noncannabinoid receptors expressed in neural, immune, and epithelial cells. Notably, these receptors are also present in osteogenic and progenitor cells, suggesting that CBD may influence bone metabolism and regeneration. Recent preclinical studies have reported that CBD enhances osteoblastic differentiation, angiogenesis, and matrix mineralization, highlighting its potential as a bioactive molecule for bone tissue engineering. Within the dental field, such properties open new perspectives for the development of CBD-based biomaterials aimed at improving osseointegration, soft tissue healing, and the overall biological performance of implantable devices. Accordingly, this review aims to provide a comprehensive overview of the pharmacological and molecular mechanisms underlying the effects of CBD on wound healing and bone regeneration. Furthermore, it discusses dose–response relationships, delivery routes, formulation strategies, and the current legal and regulatory frameworks influencing CBD translation into clinical dental applications. These insights may support the rational design of next-generation bioactive materials incorporating CBD for oral and maxillofacial regenerative therapies.

## Graphical abstract

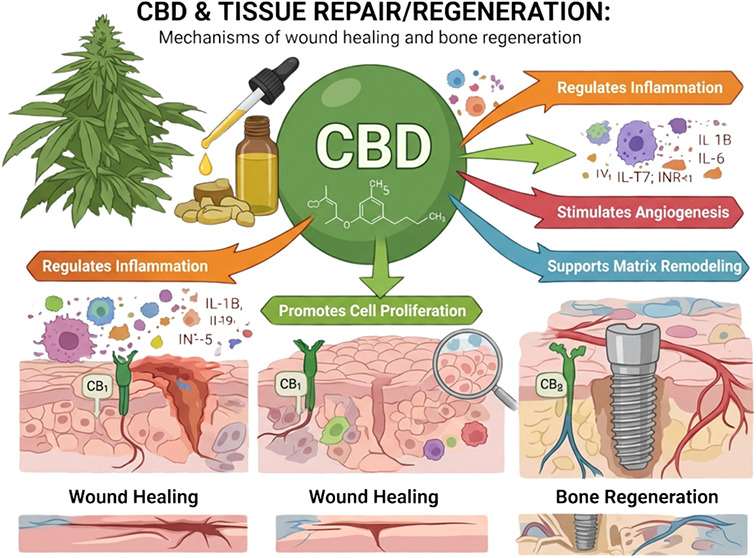

## 1 Introduction

Tissue injury initiates a highly orchestrated and dynamic sequence of cellular, molecular, and biochemical events designed to restore structural integrity and functional homeostasis.^1,2^ Successful wound healing relies on the precise coordination of four interdependent and overlapping phases, hemostasis, inflammation, proliferation, and remodeling or resolution, each governed by a complex interplay of immune cells, cytokines, and growth factors.^3^ The timely and balanced transition between these phases is critical for effective repair. However, dysregulation of the immune response can disrupt this sequence, leading to chronic inflammation, and impaired regeneration.^4^

In the skeletal system, excessive or unresolved inflammation exerts particularly detrimental effects. An exacerbated inflammatory milieu can stimulate osteoclastogenesis, enhancing bone resorption and compromising the stability of the surrounding tissue.^4^ Bone defects frequently arise from trauma, infection, tumor resection, or chronic inflammatory diseases and may surpass the intrinsic regenerative capacity of the host.^5^ In extensive or complex lesions, spontaneous healing becomes unfeasible due to persistent inflammation, inadequate vascularization, and the absence of osteogenic or osteoinductive cues required for new bone formation.^5^ Such critical-size defects, defined as defects incapable of spontaneous healing even after surgical stabilization, require adjunctive therapeutic strategies, including autologous bone grafting or engineered biomaterials to restore structural and functional continuity.^6^ Consequently, bone regeneration remains one of the most demanding challenges in regenerative medicine, influenced by anatomical location, defect size, and local biological conditions.^7^

To counteract inflammation-associated tissue destruction and promote regenerative healing, a variety of therapeutic strategies have been developed.^8,9^ Conventional interventions include the debridement of necrotic tissue, rigorous infection control, surgical revascularization, and bone grafting procedures employing autologous, allogeneic, or synthetic substitutes.^8,9^ Although clinically effective, these approaches are frequently limited by donor-site morbidity, immune incompatibility, infection risk, and suboptimal osteogenic potential. As a result, increasing attention has been directed toward alternative or complementary strategies, particularly those involving bioactive natural compounds and biomaterials endowed with intrinsic regenerative and immunomodulatory properties.^10–12^

Among these emerging therapeutic approaches, the medicinal potential of *Cannabis sativa* L. has attracted renewed scientific interest. Historical evidence documents its therapeutic application as early as 1550 BCE in ancient Egyptian papyri.^13^ The plant’s remarkable ecological adaptability has enabled its widespread cultivation across diverse geographic regions, leading to the identification of more than 450 bioactive constituents, including approximately 70 phytocannabinoids.^14,15^ Structurally, these compounds share a conserved dibenzopyran core linked to a hydrophobic alkyl side chain, a configuration that confers marked lipophilicity and facilitates their interaction with membrane-bound receptors^16^ (Figure 1).

**Figure 1:**
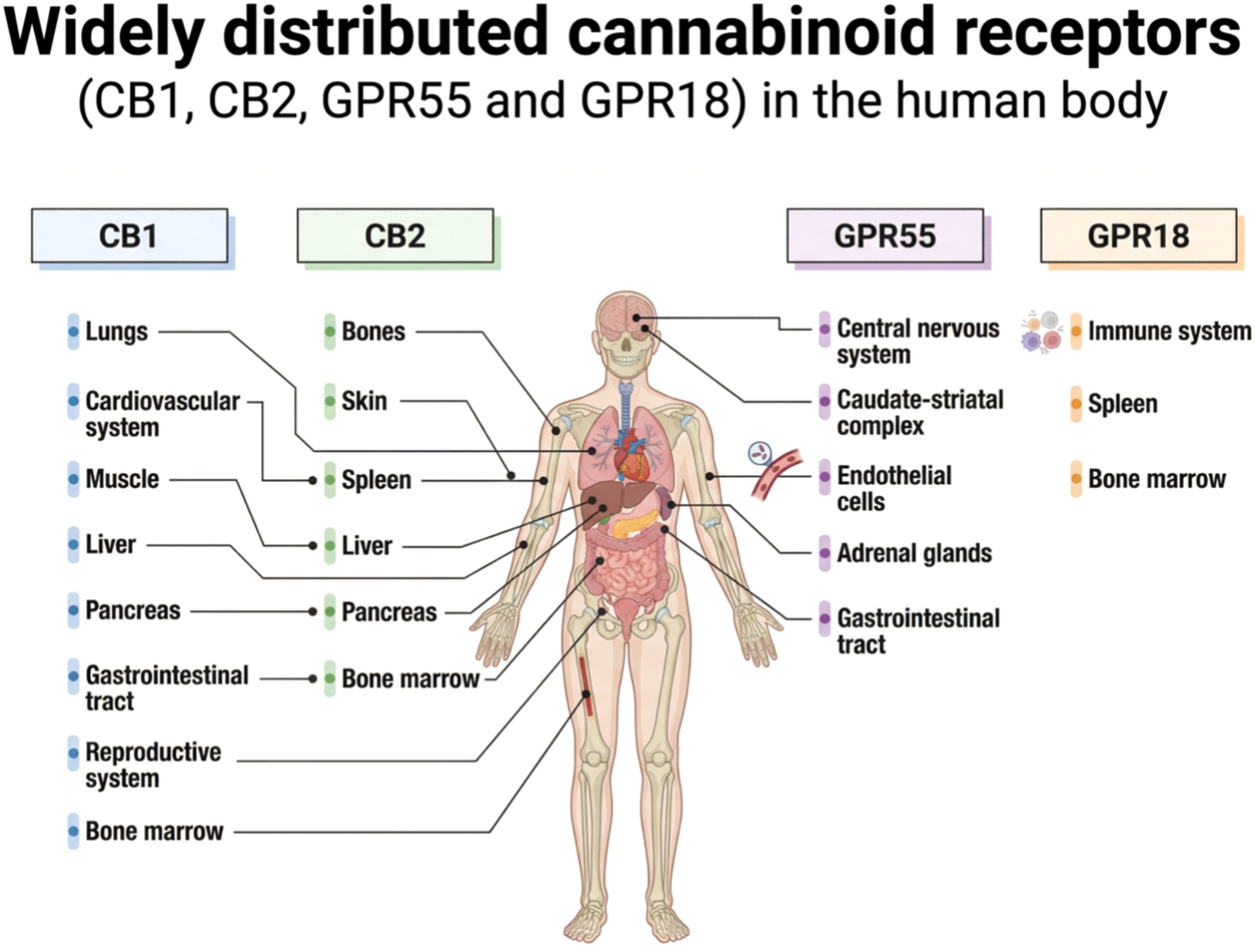
Widely distributed cannabinoid receptors (CB1, CB2, GPR55 and GPR18) in the human body. Created in BioRender. de Avila, E. (2026) https://BioRender.com/u8xbeyf. *License: RC29JN1JK5*.

The biological effects of phytocannabinoids are primarily mediated through the endocannabinoid system (ECS), a complex signaling network composed of endogenous ligands, metabolic enzymes, and cannabinoid receptors CB_1_ and CB_2_.^17^ CB_1_ receptors are predominantly expressed in the central and peripheral nervous systems but are also detected in peripheral tissues, including bone, adipose tissue, myocardium, reproductive organs, and retina.^18^ CB_2_ receptors are more abundant in immune cells, yet they are also present in the liver, gastrointestinal tract, brain, and skeletal tissues.^19^ Importantly, both CB_1_ and CB_2_ receptors have been identified in bone marrow–derived stromal cells and within the bone immune microenvironment.^20^ Experimental evidence indicates that pharmacological activation or genetic modulation of these receptors influences osteoblast and osteoclast activity, attenuates pathological bone turnover, and regulates remodeling dynamics^20^ (Figure 1).

Beyond this classical CB_1_/CB_2_ framework, increasing evidence supports the existence of an expanded endocannabinoid network that includes noncanonical receptors operating through CB_1_/CB_2_-independent pathways.^21^ Among these, GPR18 and GPR55 have emerged as putative cannabinoid-responsive receptors capable of being modulated by endocannabinoids, phytocannabinoids, and synthetic ligands. GPR18, also referred to as the N-arachidonoylglycine (NAGly) receptor, is predominantly expressed in immune cells and plays a role in neutrophil migration, macrophage phenotype modulation, and lymphocyte maturation.^22^ It is also highly expressed in microglial cells, which are key components of the central nervous system immune network, where it participates in chemotactic signaling and leukocyte recruitment to sites of injury.^19^ Cannabidiol (CBD) has been described as a modulator of orphan receptors, including GPR18, acting as an antagonist or inverse agonist under certain conditions. These interactions may partly explain CBD’s anti-inflammatory properties independent of CB_1_ activation, thereby avoiding the psychotropic effects associated with Δ^9^-tetrahydrocannabinol (Δ^9^-THC).^19^

GPR55, in contrast, signals primarily via *G*_12/13_ and *G*_q_ proteins rather than Gi-coupled mechanisms typical of CB receptors. This distinction leads to activation of intracellular pathways such as RhoA and calcium mobilization, processes involved in cell proliferation, migration, and angiogenesis.^23^ GPR55 expression has been identified in multiple central nervous system regions, including the caudate-striatal complex, as well as in peripheral tissues such as endothelial cells, adrenal glands, and the gastrointestinal tract.^24^ Its wide distribution suggests involvement in innate and adaptive immune regulation.^25^ Collectively, these findings underscore the complexity of cannabinoid-related signaling and reinforce the concept that phytocannabinoid effects extend beyond the classical CB_1_/CB_2_ paradigm, engaging a broader receptor repertoire relevant to immune modulation and tissue homeostasis.

Among phytocannabinoids, Δ^9^-THC and cannabidiol (CBD) are the most extensively studied due to their distinct pharmacological profiles.^26^ Δ^9^-THC, isolated in 1964, is the principal psychoactive constituent of the plant and exerts its central effects primarily via CB_1_ activation.^27,28^ In contrast, CBD is nonpsychoactive and exhibits low affinity for CB_1_ and CB_2_ receptors. Instead, it modulates the ECS indirectly and interacts with additional molecular targets, including TRPV1, PPARγ, and 5-HT1A receptors.^29^

CBD has garnered substantial attention due to its wide range of reported therapeutic effects, encompassing anti-inflammatory, antioxidant, neuroprotective, and regenerative properties.^30,31^ These pleiotropic actions suggest that CBD may act as a multimodal agent capable of modulating several signaling pathways relevant to tissue homeostasis and repair. From a regenerative perspective, an ideal bioactive compound should attenuate inflammation and oxidative stress, exhibit antimicrobial activity, and promote cellular proliferation, matrix deposition, and angiogenesis.^32,33^ In this context, preclinical evidence has demonstrated the pro-healing capacity of CBD. For instance, Yan et al.^34^ reported that topical CBD administration enhanced wound closure and vascularization through the upregulation of vascular endothelial growth factor (VEGF) in granulation tissue. Additional studies have shown that CBD modulates inflammatory responses, promotes collagen synthesis, and stimulates neovascularization, thereby supporting its potential role in tissue regeneration.

Despite this promising evidence, the precise molecular and cellular mechanisms underlying CBD-mediated tissue remodeling remain incompletely understood. Considering the pivotal role of inflammation in regulating bone remodeling and the detrimental effects of excessive immune activation on bone integrity, the immunomodulatory functions of CBD are of particular relevance.^35^ Notably, Li et al.^35^ demonstrated that CBD reduced the mRNA expression of proinflammatory cytokines such as TNF-α and IL-6 in lipopolysaccharide-stimulated bone marrow mesenchymal stem cells, while concurrently enhancing the expression of osteogenic markers, including Runx2, alkaline phosphatase (ALP), and osteocalcin (OCN).

Given the multifactorial nature of wound healing and bone regeneration, elucidating the biological mechanisms through which natural compounds like CBD modulate inflammation and stimulate reparative processes is crucial for advancing translational regenerative strategies. The exploration of such bioactive molecules offers novel opportunities to overcome the limitations of conventional therapies and improve clinical outcomes. Accordingly, this review aims to provide a comprehensive analysis of the chemical and biological mechanisms underlying the effects of CBD in wound healing and bone regeneration, with particular emphasis on its immunomodulatory and osteogenic properties. Furthermore, it addresses key considerations regarding dosage, administration routes, formulation strategies, and regulatory aspects to optimize its therapeutic potential in regenerative medicine.

## 2 Natural versus Impaired Course of Wound Healing

Wound healing follows a tightly regulated physiological cascade involving overlapping cellular and molecular events that collectively restore tissue integrity and function.^36,37^ Under normal conditions, this process proceeds in a coordinated manner through the sequential phases of hemostasis, inflammation, proliferation, and remodeling, ultimately culminating in the re-establishment of tissue homeostasis. However, the persistence of inflammatory stimuli, microbial contamination, ischemia, or metabolic dysfunction can disrupt this orderly sequence, leading to delayed, chronic, or pathological healing outcomes. Such disturbances often result in excessive inflammation, impaired cell migration and proliferation, and aberrant extracellular matrix deposition, all of which compromise functional tissue restoration.

A clear understanding of the fundamental differences between physiological (natural) and dysregulated (non-natural or impaired) healing processes is essential for the rational design of therapeutic interventions targeting tissue regeneration. This distinction provides a critical conceptual framework for evaluating the pharmacological potential of bioactive compounds, such as cannabidiol (CBD), in modulating the wound-healing response. Prior to developing any therapeutic formulation aimed at enhancing repair in acute or chronic wounds, it is imperative to establish a comprehensive understanding of the molecular mechanisms that distinguish the natural from the impaired course of healing (Figure 2).

**Figure 2:**
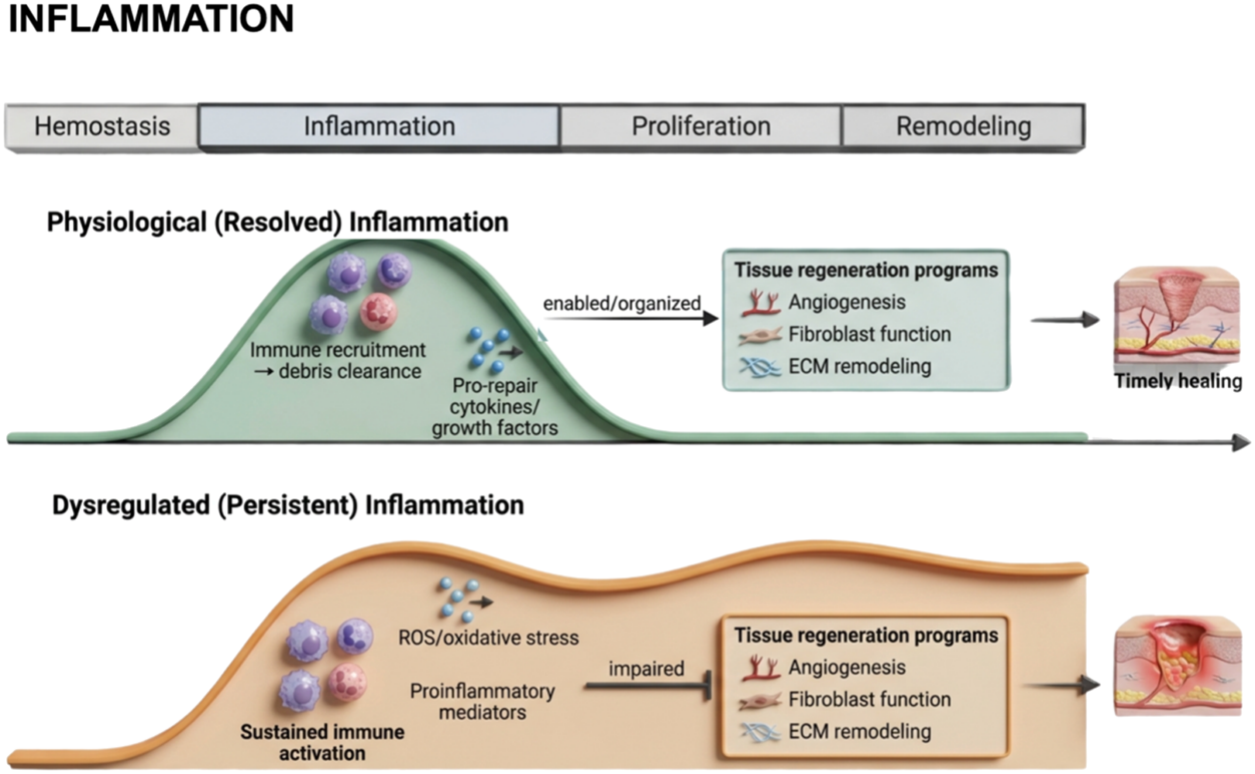
Schematic overview of the natural versus dysregulated course of inflammation during wound healing. Under physiological conditions, wound healing proceeds through a tightly coordinated sequence of overlapping phases, hemostasis, inflammation, proliferation, and remodeling, that collectively restore tissue structure and function. Controlled inflammation plays a pivotal role in initiating repair by recruiting immune cells, clearing debris, and releasing cytokines and growth factors that guide subsequent regenerative events. In contrast, when inflammatory stimuli persist or become excessive, this delicate balance is disrupted, leading to prolonged immune activation, oxidative stress, and the overproduction of proinflammatory mediators. Such dysregulation impairs angiogenesis, fibroblast function, and extracellular matrix remodeling, ultimately resulting in delayed or chronic wound healing. The illustration highlights the key cellular and molecular differences between the natural and pathological inflammatory trajectories and their respective outcomes in tissue repair. Created in BioRender. de Avila, E. (2026) https://BioRender.com/lmp1tiu. *License: YM29ED40J1*.

### 2.1 Natural Course of Wound Healing: From Hemostasis to Remodeling

The physiological (natural) course of wound healing is a highly orchestrated process that progresses through four dynamic and overlapping phases: hemostasis, inflammation, proliferation, and remodeling^38^ (Figure 3). Hemostasis, the immediate response to tissue injury, involves vascular constriction, platelet aggregation, and fibrin clot formation, establishing a provisional matrix and limiting blood loss. Inflammation follows, characterized by the sequential recruitment and activation of neutrophils and macrophages, which remove debris and pathogens while releasing cytokines and growth factors that orchestrate subsequent regenerative events. The proliferative phase entails fibroblast proliferation, extracellular matrix deposition, angiogenesis, and epithelial migration, collectively restoring tissue architecture. Finally, remodeling (maturation) involves the reorganization of collagen fibers, resolution of neovasculature, and restoration of tissue tensile strength, culminating in functional repair.

**Figure 3:**
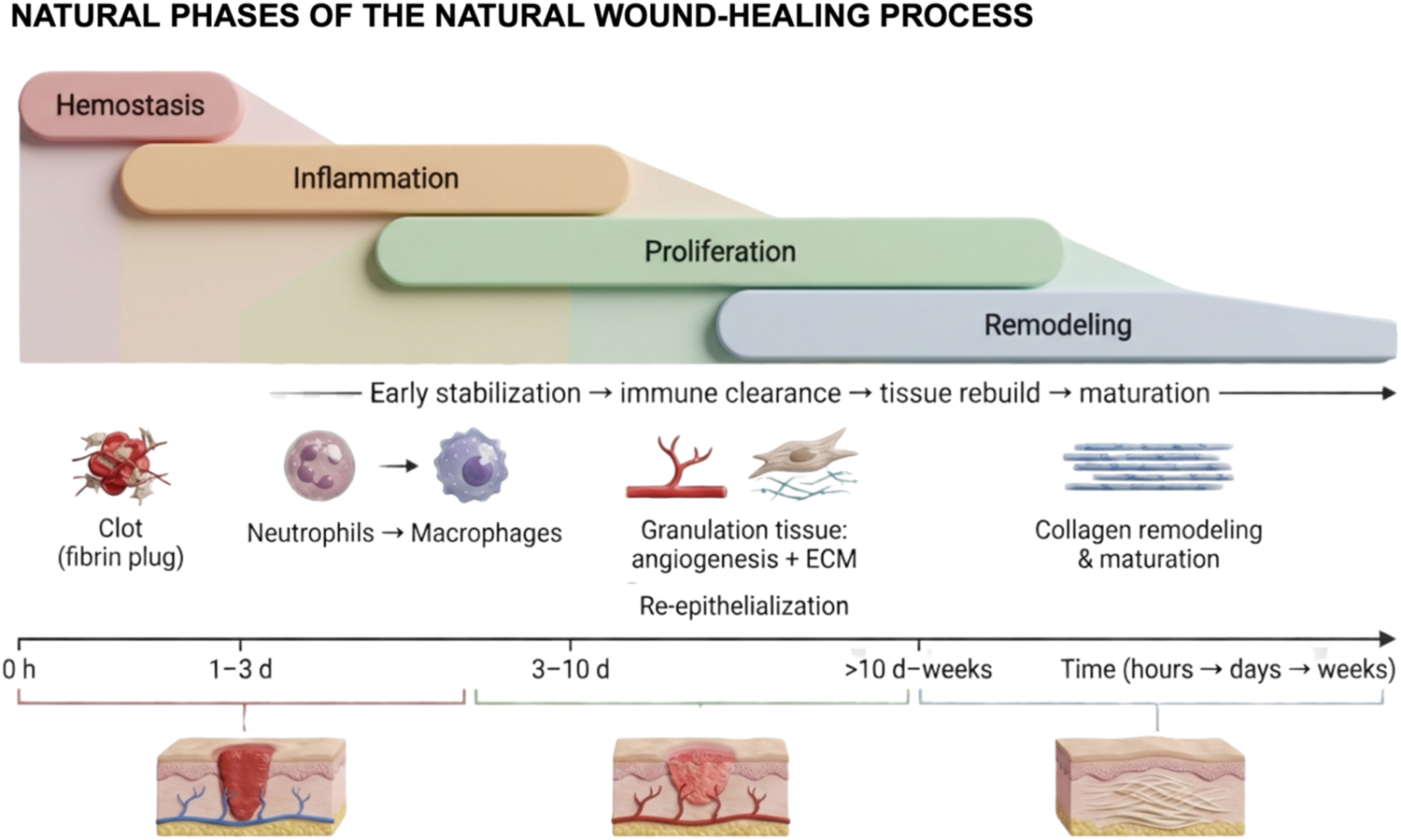
Schematic representation of the overlapping and sequential phases of the natural wound-healing process. The figure illustrates the dynamic progression from hemostasis and inflammation to proliferation and remodeling, highlighting the temporal and cellular interplay among key events such as clot formation, immune cell infiltration, angiogenesis, extracellular matrix deposition, and tissue maturation. Created in BioRender. de Avila, E. (2026) https://BioRender.com/dlw1s68. *License: VQ29EDBNCG*.

This well-regulated sequence provides the benchmark for assessing the therapeutic potential of bioactive compounds, such as CBD. By modulating inflammatory responses, promoting fibroblast and endothelial cell activity, and facilitating the transition from inflammation to proliferation and remodeling, CBD may enhance the efficiency and quality of tissue repair, particularly in chronic or nonhealing wounds. Understanding the natural course of healing is therefore essential for interpreting CBD’s regenerative mechanisms and optimizing its application in tissue engineering and regenerative medicine.

The initial phase of wound healing, hemostasis, functions as a protective mechanism that limits blood loss and establishes a provisional matrix for subsequent tissue repair.^39^ Immediately following injury, vasoconstrictive mediators, including endothelin, released by damaged endothelium, and systemic factors such as epinephrine, stimulate contraction of vascular smooth muscle, transiently reducing blood flow at the injury site.^40^ Concurrently, mediators such as bradykinin and fibrinopeptides initiate the coagulation cascade, leading to platelet aggregation and the formation of a primary hemostatic plug.^38^

Coagulation proceeds via two principal pathways: the extrinsic pathway, triggered by tissue factor exposed at the site of vascular injury, and the intrinsic pathway, activated by contact between blood components and negatively charged surfaces, such as exposed collagen or matrix proteins.^38,41^ Both pathways converge on the activation of factor X, initiating the common pathway that leads to the conversion of prothrombin into thrombin.^42^ Thrombin subsequently catalyzes the transformation of fibrinogen into fibrin, forming an insoluble network that stabilizes the platelet plug and establishes a secondary hemostatic structure, often referred to as the provisional matrix.^38^

This fibrin-based matrix serves as a scaffold for cellular infiltration, initiating the inflammatory phase of wound healing. Neutrophils are among the first immune cells recruited to the wound site, where they perform essential functions including pathogen clearance, phagocytosis of debris, and secretion of proinflammatory mediators to orchestrate the subsequent reparative response.^43^ The migration of inflammatory cells is guided by chemotactic signals generated by tissue-resident cells in response to damage-associated molecular patterns (DAMPs) and pathogen-associated molecular patterns (PAMPs), alerting neutrophils in the bone marrow to the site of injury and facilitating their directed movement into the wound.^43,44^

Upon arrival at the injury site, neutrophils release cytotoxic granules containing proteolytic enzymes and reactive oxygen species, which directly target and eliminate invading pathogens.^38^ They also perform phagocytosis via surface antigen receptors, engulfing and degrading microbial and cellular debris.^36^ Concurrently, macrophages infiltrate the wound, initially adopting a pro-inflammatory (M1) phenotype, characterized by the production of cytokines such as interleukin (IL)-6, tumor necrosis factor-α (TNF-α), and IL-1β, thereby amplifying the antimicrobial response.^45^ Macrophages also contribute to resolution of inflammation by phagocytosing senescent neutrophils that fail to return to the bone marrow, effectively terminating the inflammatory phase.^38,46^ Following this, M1 macrophages undergo phenotypic switching to the anti-inflammatory, tissue-repair-promoting M2 phenotype, which supports angiogenesis, extracellular matrix (ECM) deposition, and immunoregulation.^45^

The proliferative phase is initiated with the replacement of the fibrin clot by granulation tissue and the restoration of vascular supply via angiogenesis, ensuring delivery of oxygen and nutrients to the regenerating tissue.^38,47^ This phase involves coordinated activity of keratinocytes, fibroblasts, macrophages, and endothelial cells, aimed at reestablishing a functional tissue barrier.^48^ Endothelial cells respond to hypoxia by upregulating vascular endothelial growth factor (VEGF), proliferating, and forming new capillary networks, thereby facilitating nutrient and oxygen delivery essential for effective tissue repair.^38,49^ Meanwhile, fibroblasts, stimulated by signals from platelets, endothelial cells, and M2 macrophages, deposit a provisional extracellular matrix, predominantly composed of type III collagen. A subset of fibroblasts differentiates into myofibroblasts, which generate contractile forces that reduce wound size and contribute to tissue integrity.^50^

The remodeling (maturation) phase represents the final stage of wound healing, during which the granulation tissue is reorganized into mature, functional tissue. M2 macrophages transition to a phenotype (M2c) involved in ECM turnover, releasing matrix metalloproteinases (MMPs) that degrade excess collagen and remodel the provisional matrix.^45^ During this phase, type III collagen is gradually replaced by type I collagen, restoring the tensile strength of the tissue.^38,51^ Additional structural components, such as elastin, are also reincorporated into the regenerating tissue, contributing to the functional and mechanical properties of the scar.^45^ Together, these tightly regulated events ensure the successful resolution of the wound and the restoration of tissue homeostasis.

### 2.2 Non-Natural Course of Wound Healing: Persistence of Inflammatory Stimuli

In contrast to the tightly regulated sequence of cellular, humoral, and molecular events that characterize physiological wound healing, certain pathological conditions disrupt this process, leading to impaired regeneration. Such wounds may either form excessive scar tissue or develop into chronic lesions that exhibit minimal reduction in size (typically <40–50%) and fail to heal effectively^52^ (Figure 4). In these scenarios, the anti-inflammatory and immunomodulatory properties of cannabidiol (CBD) may provide therapeutic benefit by attenuating persistent inflammation and promoting progression toward tissue repair.

**Figure 4:**
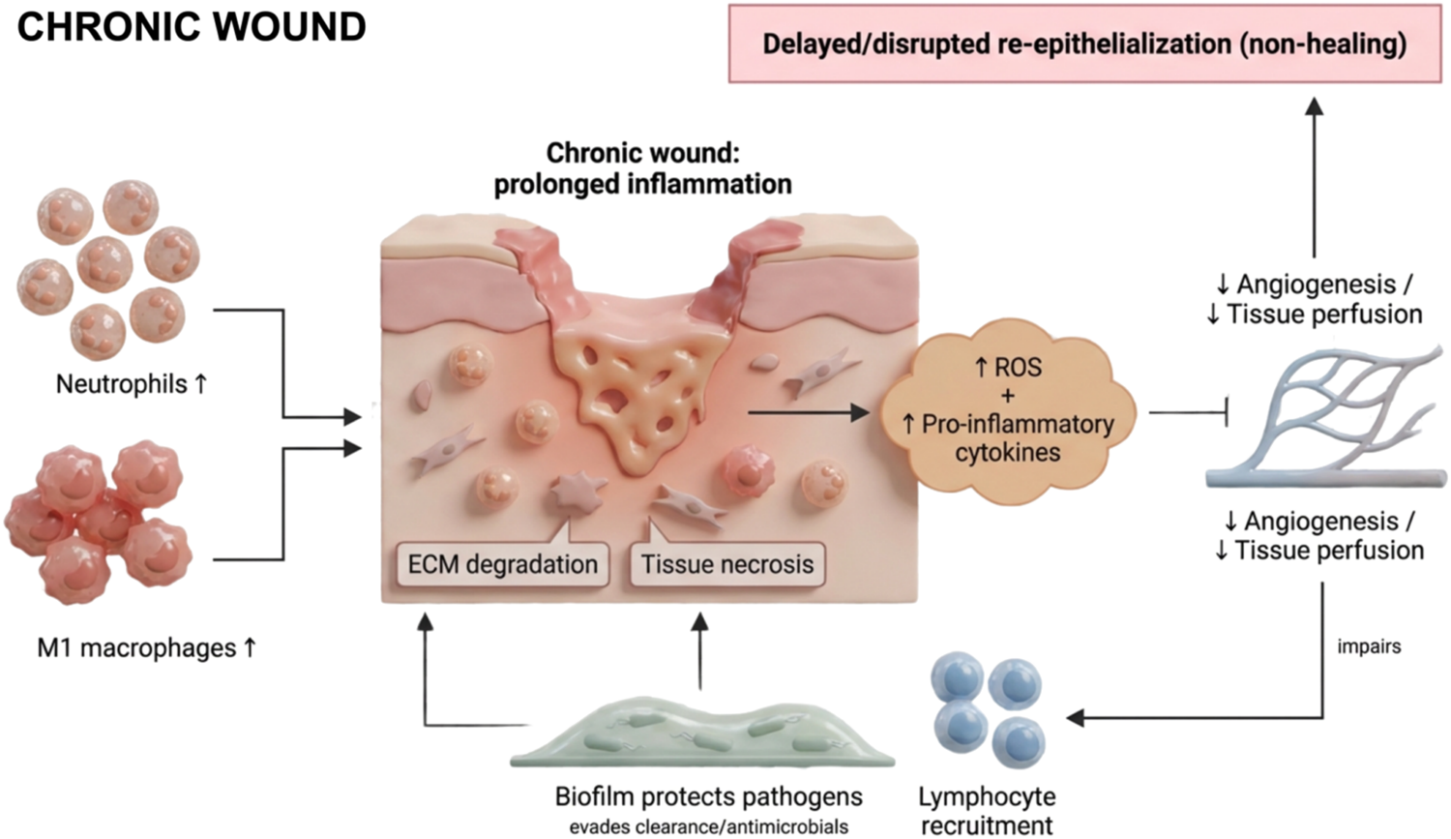
Schematic representation of the pathological course of a chronic wound. Chronic wounds are characterized by a prolonged and amplified inflammatory phase, with excessive infiltration of neutrophils and pro-inflammatory (M1) macrophages. This sustained immune activation leads to elevated production of reactive oxygen species (ROS) and pro-inflammatory cytokines, which collectively impair angiogenesis and tissue perfusion, contributing to necrosis and delayed or disrupted re-epithelialization. Persistent inflammation is further reinforced by lymphocyte recruitment and the presence of biofilms, which protect pathogens from host immune responses and antimicrobial therapies. The figure illustrates the key cellular and molecular features that perpetuate chronic inflammation, ECM degradation, and impaired tissue repair in nonhealing wounds. Created in BioRender. de Avila, E. (2026) https://BioRender.com/eedgtyc. *License: TF29ED7GCB*.

Chronic wound development is influenced by intrinsic factors, including age, malnutrition, diabetes, and immunosuppression, as well as extrinsic factors such as local temperature, humidity, and infection.^53,54^ Pathologically, chronic wounds are characterized by sustained inflammation, recurrent infections, necrosis, impaired re-epithelialization, reduced angiogenesis, and excessive production of reactive oxygen species (ROS).^55^ Dysregulation may begin as early as the hemostatic phase; for example, hypercoagulable states can lead to thrombosis, which occludes blood vessels, restricts oxygen delivery, and induces tissue ischemia. Similarly, conditions such as hyperglycemia in diabetic individuals can elevate ROS levels, which, beyond their physiological role in vasoconstriction during hemostasis and vasodilation during proliferation, disrupt endothelial cell function, impair angiogenesis, and promote apoptosis.^38,56^

Persistent inflammation is the primary driver of the non-natural healing trajectory.^54^ Chronic wounds are marked by prolonged infiltration of myeloid cells—including neutrophils, monocytes, and macrophages—into the late inflammatory phase.^57^ Within these wounds, there is an imbalance between pro-inflammatory (M1) and anti-inflammatory (M2) macrophages, with insufficient resolution of inflammation.^58^ Impaired clearance of apoptotic neutrophils exacerbates the inflammatory milieu, sustaining high levels of cytokines such as TNF-α and IL-1β, which perpetuate tissue degradation.^59,60^ Additionally, macrophage-derived matrix metalloproteinases (e.g., MMP-2 and MMP-9) degrade extracellular matrix components, further delaying the proliferative phase.^59^ Fibrocytes, a subset of macrophage-derived cells responsible for ECM deposition, may contribute to excessive fibrosis when dysregulated.^61^

Crosstalk between immune cells and nonhematopoietic cells, such as keratinocytes, is also disrupted in chronic wounds. Aberrant expression of microRNAs, including miR-34a/c, miR-203, miR-19a/b, and miR-20a, alters keratinocyte-mediated immune regulation, delays re-epithelialization, and amplifies inflammation via upregulation of the NF-κB pathway, resulting in increased pro-inflammatory cytokine and chemokine production.^35,62^ Collectively, these failures in immune coordination prevent proper tissue repair, resulting in chronic wounds or pathological scarring.^61,63^

Moreover, microbial colonization plays a central role in perpetuating chronic inflammation and impairing tissue repair. A systematic review encompassing 185 chronic wounds reported that 78.2% contained polymicrobial biofilms, which provide structural and biochemical protection to pathogens against host immune defenses and antimicrobial therapies.^63,64^ In addition, coinfection with microorganisms such as Candida spp. and *Porphyromonas gingivalis* has been shown to inhibit cellular migration *in vitro*, underscoring the contribution of complex microbial communities to delayed healing, particularly in oral mucosal lesions following cancer therapy.^65^

In contrast, the wound healing process differs substantially between cutaneous and oral mucosal tissues. Experimental models demonstrate that oral mucosal wounds exhibit reduced pro-fibrotic signaling and an expansion of fibroblast populations, which collectively promote earlier re-epithelialization, accelerated wound closure, and diminished scar formation compared with comparable skin lesions.^66,67^ Interleukin-1 (IL-1) signaling appears to play a tissue-specific role in the oral mucosa, being essential for efficient healing and protection of open wounds from bacterial invasion, while exerting comparatively limited effects on cutaneous wound closure under similar conditions.^68^ Supporting the concept that oral tissues are primed for a heightened inflammatory responsiveness due to continuous microbial exposure, ex vivo gingival biopsies have been shown to secrete higher levels of pro-inflammatory cytokines, including IL-6, IL-8, IL-1β, IL-10, and TNF-α, than skin biopsies.^69^ Furthermore, salivary secretions play a critical role in oral tissue repair, as evidenced by hyposalivation models demonstrating delayed palatal wound healing, thereby highlighting the importance of locally derived soluble factors in mucosal regeneration.^70^ Collectively, these findings emphasize the distinct immunobiological environment of oral tissues and its implications for wound healing dynamics, particularly in the context of microbial burden and local regulatory factors.

## 3 Biological and Chemical Mechanisms of Cannabidiol in Wound Healing

CBD, a nonpsychoactive phytocannabinoid isolated from *C. sativa* L., has attracted considerable attention for its broad therapeutic potential, encompassing anti-inflammatory, analgesic, antioxidant, and pro-regenerative effects.^71^ While early cannabinoid research predominantly addressed psychoactive properties, emerging evidence since the early 2000s highlights CBD’s capacity to modulate immune responses, cellular signaling pathways, and tissue repair mechanisms.^72^ In the context of wound healing, CBD exerts effects across multiple overlapping phases, including the inflammatory, proliferative, and remodeling stages, by regulating immune cell function, oxidative stress, extracellular matrix deposition, and angiogenesis.

### 3.1 Cannabidiol and the Inflammatory Phase of Wound Healing

While inflammation is essential for initiating the natural wound-healing process^73^, its dysregulation can shift healing toward a nonphysiological trajectory, characterized by chronic inflammation, delayed repair, and fibrosis.^74^ This underscores the therapeutic importance of modulating, rather than suppressing, the inflammatory response to promoting effective tissue regeneration.

Cannabidiol (CBD) offers a promising strategy for controlling excessive inflammation due to its multifaceted biological and chemical mechanisms. Unlike classical anti-inflammatory agents, which typically act via cyclooxygenase inhibition, CBD does not entirely suppress inflammation; instead, it modulates the response by reducing excessive cytokine release, limiting oxidative stress, and attenuating aberrant immune cell infiltration, thereby facilitating the resolution phase of healing.^75,76^

Mechanistically, cannabidiol (CBD) exerts its biological effects primarily through inhibition of the nuclear factor kappa B (NF-κB) signaling pathway, a key regulator of pro-inflammatory gene interleukin-1 receptor antagonist (IL-1Ra) and selected cytokines^75,77^ (Figure 5). *In vitro* studies further elucidate these molecular mechanisms. For example, Sangiovanni et al.^76^ demonstrated in HaCaT keratinocytes that CBD significantly reduced the release of vascular endothelial growth factor (VEGF), a central mediator of angiogenesis, and downregulated matrix metalloproteinase-9 (MMP-9), an enzyme critically involved in extracellular matrix degradation, in a dose-dependent manner. Additionally, CBD attenuated tumor necrosis factor-α (TNF-α) expression via NF-κB inhibition, highlighting its ability to modulate pro-inflammatory signaling without completely suppressing physiological inflammatory responses.^76^ Beyond canonical pathway regulation, CBD also interacts with targets within the expanded endocannabinoid system, including GPR18. Emerging evidence indicates that CBD may function as a partial agonist of GPR18, promoting activation of the MAPK p44/42 signaling cascade, which is implicated in cellular processes essential for tissue repair and regeneration, such as proliferation, migration, and the regulation of apoptosis and autophagy.^78^ Collectively, these findings underscore the pleiotropic signaling profile of CBD and its potential therapeutic relevance in modulating inflammation while supporting regenerative tissue outcomes. Collectively, these findings suggest that CBD acts as a regulatory modulator of inflammation, creating a favorable environment for the subsequent proliferative and remodeling phases of wound healing.

**Figure 5:**
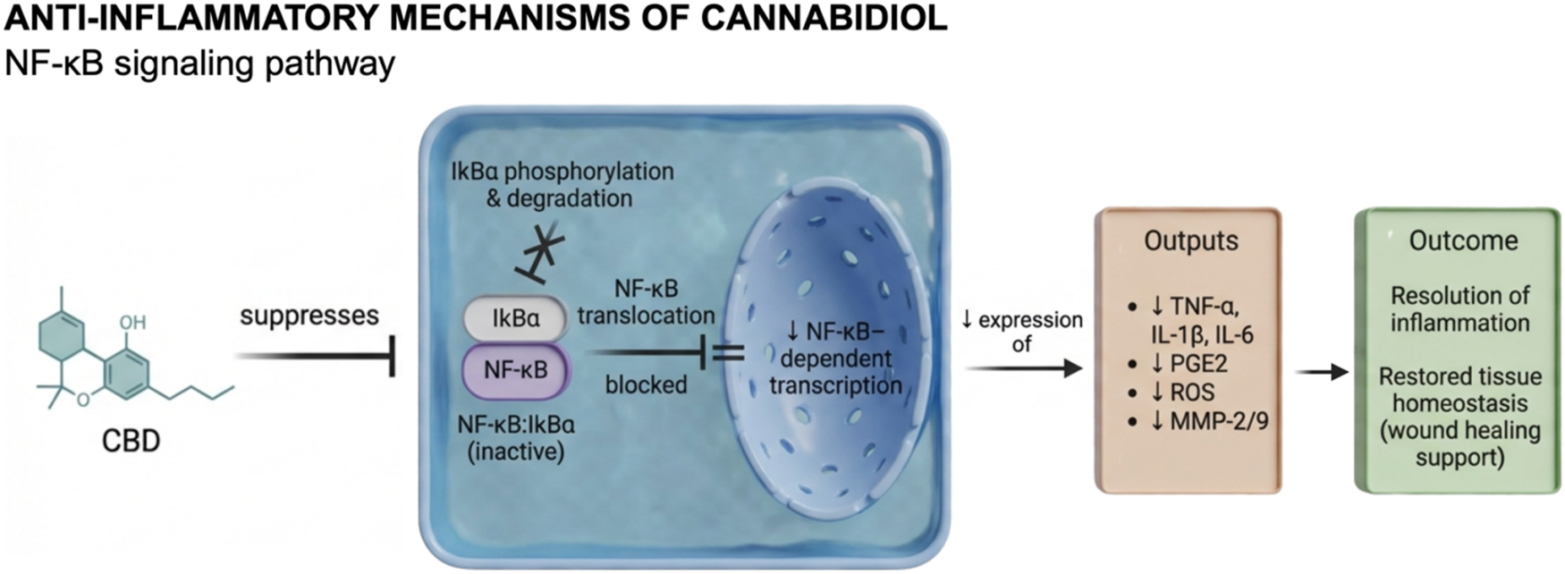
Schematic representation of the anti-inflammatory mechanisms of cannabidiol (CBD) through modulation of the NF-κB signaling pathway. CBD exerts multifactorial anti-inflammatory effects by interfering with key molecular events that control the transcription of pro-inflammatory genes. Mechanistically, CBD suppresses the phosphorylation and subsequent degradation of IκBα, thereby preventing NF-κB translocation from the cytoplasm to the nucleus. This results in the downregulation of NF-κB–dependent gene expression, including tumor necrosis factor-α (TNF-α), interleukin-1β (IL-1β), interleukin-6 (IL-6), and prostaglandin E2 (PGE2). In addition, CBD modulates oxidative stress by reducing reactive oxygen species (ROS) generation and inhibits the expression of matrix metalloproteinases (MMP-2 and MMP-9), which are responsible for extracellular matrix degradation. Collectively, these mechanisms contribute to the resolution of inflammation and restoration of tissue homeostasis, highlighting CBD’s potential as a therapeutic modulator in both acute and chronic wound environments. Created in BioRender. de Avila, E. (2026) https://BioRender.com/11yguh7. *License: KS29EDEUJ8*.

Kongkadee et al.^75^ and Tran et al.^79^ corroborated these findings by demonstrating that CBD downregulates pro-inflammatory mediators, particularly TNF-α and IL-1β, in macrophage-like and monocyte-derived cells. Both studies showed that CBD effectively inhibits phosphorylation of the p65 subunit of NF-κB, a critical step for its nuclear translocation and activation of inflammatory gene transcription. Specifically, Kongkadee et al.^75^ identified CBD as the most potent bioactive compound within hemp extract, showing that treatment with 1 μg/mL maximally suppressed TNF-α production in RAW 264.7 macrophages. Moreover, CBD at concentrations ranging from 5 to 50 μg/mL significantly decreased IL-1β secretion, further confirming its dose-dependent immunomodulatory potential. Similarly, Tran et al.^79^ reported that treatment of THP-1 cells with 6.6 μM CBD markedly reduced IL-6 and TNF-α production, concomitant with inhibition of IκB degradation—an inhibitory protein that prevents NF-κB activation. In agreement with these findings, Kozela et al.^80^ demonstrated that CBD impedes phosphorylation of the NF-κB p65 subunit, thereby preventing its nuclear translocation and subsequent transcription of pro-inflammatory cytokines (Figure 5).

Evidence from in vivo studies further substantiates the anti-inflammatory properties of CBD. Klein et al.^81^ investigated the effects of CBD on oral wound healing in Wistar rats and observed significantly lower inflammatory scores in CBD-treated animals compared to vehicle controls by the third day postinjury, indicating accelerated resolution of inflammation. Likewise, Genovese et al.^77^ demonstrated that oral administration of CBD (10 mg/kg) reduced endometriosis-associated inflammation in Sprague–Dawley rats. This was characterized by increased IκB-α expression, decreased cytosolic cyclooxygenase-2 (COX-2) levels, and reduced nuclear localization of NF-κB within lesion tissues. Moreover, the treatment led to reduced levels of TNF-α, IL-1β, and prostaglandin E2 (PGE2) in the peritoneal environment, collectively confirming CBD’s ability to modulate both systemic and localized inflammatory responses.

Expanding on these findings, Zhou et al.^82^ recently developed a multifunctional hydrogel incorporating CBD to promote the repair of radiation-induced and cutaneous wounds. The hydrogel markedly reduced inflammation, enhanced collagen deposition, and accelerated tissue regeneration. Mechanistically, CBD treatment was associated with modulation of key cytokines and chemokines, including IL-6, IL-17A, IL-22, CCL3, and CCL11, which are closely linked to the regulation of macrophage polarization and attenuation of chronic inflammatory signaling cascades. The authors proposed that these effects highlight CBD’s capacity to promote a shift toward a pro-resolving immune environment, thereby facilitating wound closure and tissue remodeling.

Taken together, the current body of evidence underscores CBD’s potential as a natural bioactive compound capable of attenuating inflammation and promoting wound repair through NF-κB pathway modulation and immune homeostasis restoration. Nonetheless, further studies are warranted to optimize therapeutic dosing, elucidate biodistribution dynamics, and assess long-term safety to ensure the translational viability of CBD-based interventions in regenerative medicine.

#### 3.1.1 Cannabidiol as an Antioxidant: Mitigating Inflammation through Reduction of Oxidative Stress

Persistent inflammation is often accompanied by oxidative stress due to an imbalance between reactive oxygen species (ROS) and antioxidant defenses.^83^ High levels of ROS impair angiogenesis, damage DNA, and disrupt cellular signaling pathways essential for healing.^84^ CBD acts as a potent antioxidant by scavenging ROS and enhancing endogenous antioxidant systems such as glutathione peroxidase and superoxide dismutase (SOD).^85,86^

Antioxidant enzymes like SOD, glutathione peroxidase, and catalase help to protect the cells in the human body from harmful reactive oxygen substances.^87^ In this context, previous studies have shown the beneficial effects of CBD on the reduction of the oxidation process.^33,77,85,86^ For instance, Genovese et al.^77^ demonstrated that daily administration of CBD to Sprague–Dawley rats with endometriosis reduced oxidative stress. The thiobarbituric acid reactive substances (TBARS) assay revealed a decrease in lipid peroxidation, while levels of glutathione (GSH) and SOD activity were restored. Furthermore, Western blot analysis showed elevated expression of NADPH oxidase 1 (Nox-1) and Nox-4 in control rats, which was significantly reduced with CBD treatment.^77^

Additionally, recent studies have focused on new biomaterials loaded with CBD, including a hydrogel made from alginate-zinc infused with CBD (CBD/Alg-Zn), which demonstrated effective antioxidant activity in reducing ROS in an *in vivo* model.^86^ In another study, Chelminiak-Dudkiewicz et al.^88^ examined chitosan-based orodispersible films enriched with cannabis oil as a source of CBD on L929 mouse fibroblasts, revealing noteworthy results in the context of antioxidant activity. The antioxidant activity of pure chitosan film was modest (9.5%), but the inclusion of CBD markedly enhanced radical-scavenging potential—up to 63.2%. DPPH assays revealed CBD concentration-dependent increases in antioxidant activity: 42.5% (1% CBD), 64.8% (5% CBD), and 72.7% (10% CBD). These findings highlight the strong antioxidant capacity of CBD-infused biomaterials for potential wound healing applications.

### 3.2 Cannabidiol and the Proliferative phase of Wound Healing

As previously discussed, the proliferative phase involves granulation tissue formation, and collagen depositon to create proteins in the extracellular matrix.^73^ The presence of fibroblasts in the wound area is crucial for the formation of granulation tissue and the synthesis and deposition of collage.^89^ A study shows that CBD may also support this phase by promoting fibroblast migration and matrix remodeling without impairing cellular proliferation.^90^

Styrczewska et al.^90^ developed a flax fiber dressing infused with CBD, which demonstrated promising outcomes in promoting cell proliferation and migration *in vitro*. Although CBD did not significantly influence fibroblast or keratinocyte morphology or proliferation, it enhanced matrix metalloproteinase activity and exerted notable anti-inflammatory effects. In wound healing assays, fibroblasts treated with CBD achieved full wound coverage within 48 h, with significantly fewer unhealed areas compared to controls.^81^ Similarly, Kongkadee et al.^75^ used an *in vitro* scratch assay to evaluate CBD’s effect on wound closure. At a concentration of 0.5 μg/mL, CBD significantly enhanced wound closure at 24-, 36-, and 48 h post-treatment, supporting the potential of CBD in wound healing.^75^ Futhermore, granulation tissue formation is also critical for wound stabilization.

#### 3.2.1 Effects of Cannabidiol on Granulation Tissue Formation

During the granulation phase of wound healing, fibroblasts play a pivotal role in synthesizing and depositing extracellular matrix (ECM) components, thereby providing structural integrity to the developing tissue. Concurrently, keratinocytes at the wound margins proliferate and migrate centripetally to re-establish the epithelial barrier.^73^ Recent advances in biomaterial-based delivery systems have sought to potentiate these cellular events through the incorporation of bioactive molecules such as cannabidiol (CBD). In this context, Zheng et al.^86^ developed a CBD-loaded alginate–zinc hydrogel (CBD/Alg–Zn) and evaluated its regenerative potential in a full-thickness excisional wound model in Sprague–Dawley rats. The CBD/Alg–Zn-treated wounds exhibited markedly enhanced granulation tissue formation, accelerated re-epithelialization, and reduced inflammatory cell infiltration compared with both the Alg–Zn-only and untreated control groups. These findings indicate that CBD, when combined with zinc and alginate, exerts synergistic effects likely attributable to its anti-inflammatory and antioxidant activities, as well as its potential to modulate fibroblast proliferation and ECM remodeling. Despite these promising outcomes, the precise molecular pathways by which CBD influences fibroblast activity, collagen deposition, and keratinocyte migration during granulation remain insufficiently characterized. Thus, further mechanistic studies, particularly those integrating molecular profiling and advanced biomaterial systems, are warranted to fully elucidate CBD’s role in this critical phase of wound repair.

#### 3.2.2 Effect of Cannabidiol on Collagen Deposition

Collagen constitutes the primary structural protein of the extracellular matrix (ECM) in skin and connective tissues, providing mechanical strength, structural integrity, and a scaffold for cell adhesion and migration during tissue repair.^91,92^ The synthesis, organization, and remodeling of collagen fibers are tightly regulated processes that determine the quality and strength of the healed tissue. Dysregulation of collagen deposition may result in impaired healing or excessive scar formation, underscoring the importance of modulating this pathway for optimal wound repair.

In this context, cannabidiol (CBD), a nonpsychoactive phytocannabinoid derived from *C. sativa*, has emerged as a bioactive compound with potential regulatory effects on ECM remodeling. Zheng et al.^86^ demonstrated that topical application of a CBD-loaded alginate-zinc hydrogel (CBD/Alg-Zn) significantly enhanced collagen deposition during the wound healing process in Sprague–Dawley rats. Histological analysis using Masson’s trichrome staining revealed a denser and more organized collagen network in both CBD/Alg-Zn- and Alg-Zn-treated groups at days 7 and 14 postinjury, with the CBD-enriched hydrogel exhibiting superior performance compared to the zinc-only formulation. These findings suggest that CBD may potentiate the reparative effects of zinc and alginate by synergistically promoting fibroblast activity and ECM synthesis.

Further supporting this notion, Qi et al.^93^ investigated the osteogenic and extracellular matrix-modulatory effects of CBD in human dental pulp stem cells (hDPSCs). After 6 h of CBD exposure, there was a marked upregulation in the expression of collagen type I and II genes, indicating that CBD can stimulate the early stages of matrix protein synthesis in mesenchymal-derived cells. Type I collagen is the predominant fibrillar form found in dermal and bone tissues, while type II collagen is characteristic of cartilaginous structures, highlighting CBD’s broad influence across different tissue types.

Although the precise molecular mechanisms underlying CBD-induced collagen deposition remain incompletely understood, emerging evidence points toward the involvement of cannabinoid receptors (CB1 and CB2) and peroxisome proliferator-activated receptor γ (PPARγ) pathways, which can regulate fibroblast proliferation, differentiation, and ECM turnover. Additionally, CBD’s antioxidant and anti-inflammatory activities may create a microenvironment favorable to collagen maturation by reducing oxidative stress and pro-inflammatory cytokine release, both of which are known to impair matrix assembly.

Despite these encouraging findings, comprehensive mechanistic and translational studies are still required to confirm the direct targets of CBD in collagen biosynthesis, fiber organization, and cross-linking. Future research should integrate molecular, histological, and biomechanical analyses to elucidate how CBD influences ECM dynamics during various phases of wound healing and tissue regeneration.

#### 3.2.3 Effects of Cannabidiol on Wound Contraction

Wound contraction constitutes a fundamental component of the proliferative phase of tissue repair, contributing to wound closure through centripetal movement of the wound edges. This process is predominantly mediated by the differentiation of fibroblasts into contractile myofibroblasts, a transition that typically occurs within 10–14 days postinjury.^89^ Myofibroblasts express α-smooth muscle actin (α-SMA) within stress fibers, generating contractile forces that reorganize the collagenous matrix and promote mechanical closure of the wound bed.^47^ The fibroblast-to-myofibroblast trans differentiation is orchestrated by a complex interplay of cytokines and growth factors, including transforming growth factor-β1 (TGF-β1), platelet-derived growth factor (PDGF), and connective tissue growth factor (CTGF), which collectively regulate cytoskeletal assembly, matrix remodeling, and integrin-mediated cell–matrix interactions.^94^

Although the role of cannabidiol (CBD) in modulating wound contraction remains incompletely elucidated, growing evidence suggests it may influence fibroblast activity, ECM remodeling, and inflammatory resolution—factors indirectly linked to contractile efficiency. Gangopadhyay et al.^95^ demonstrated that a polyherbal Ayurvedic formulation containing *C. sativa* accelerated wound contraction in full-thickness excision wounds in Wistar rats, achieving a significantly smaller wound area within 6 days of treatment. While this study did not isolate CBD as the active component, the findings imply a potential cannabinoid-mediated enhancement of granulation tissue formation and matrix remodeling.

In contrast, Klein et al.^81^ evaluated the effect of pure CBD in an oral mucosal injury model in Wistar rats. Although topical CBD application did not significantly reduce lesion size on days 3 and 7, histological analyses revealed markedly reduced inflammatory cell infiltration and lower inflammatory scores at day 3. These results indicate that CBD does not inhibit inflammation outright but rather modulates its resolution, likely through suppression of excessive pro-inflammatory cytokine release (e.g., TNF-α, IL-1β) and promotion of the transition to the proliferative phase. This immunomodulatory action may indirectly favor wound contraction by establishing a microenvironment conducive to fibroblast proliferation and differentiation, even if immediate contraction effects were not evident in oral tissue.

Further insights were provided by McIver et al.,^96^ who investigated the topical application of a CBD-manuka honey formulation in equine limb wounds. Despite the known pro-healing and antimicrobial properties of manuka honey, the combination did not significantly alter wound contraction rates or overall healing time compared to controls. These findings highlight the complexity of translating rodent and in vitro data to large-animal and clinical models, where differences in skin architecture, wound tension, and pharmacokinetic profiles can markedly influence therapeutic outcomes.

At the molecular level, preliminary studies have suggested that CBD may modulate the TGF-β/Smad signaling axis, a central pathway in myofibroblast differentiation and matrix contraction. In addition, CBD’s antioxidant and endocannabinoid receptor-mediated actions—particularly through CB2 and PPARγ activation—could attenuate excessive oxidative stress and inflammatory signaling, thereby optimizing the wound microenvironment for balanced matrix remodeling and contraction.^97,98^ However, direct evidence linking CBD to α-SMA expression or mechanical wound contraction remains scarce.

Altogether, these findings suggest that CBD’s influence on wound contraction is likely indirect, mediated through its effects on inflammatory resolution, fibroblast activation, and ECM dynamics, rather than through direct stimulation of contractile mechanisms. Future research should aim to clarify these interactions through well-controlled dose–response studies, standardized delivery systems, and mechanistic analyses involving molecular markers of myofibroblast differentiation (e.g., α-SMA, vimentin, and FAK phosphorylation). Understanding these pathways will be critical to harnessing CBD’s potential as a regulator of balanced tissue repair and fibrosis prevention.

### 3.3 Cannabidiol and the Remodeling Phase of Wound Healing

The remodeling or maturation phase represents the final stage of wound healing, typically commencing 2 to 3 weeks after injury and persisting for several months. This phase is characterized by the replacement of the provisional extracellular matrix (ECM) with a mature, functionally organized matrix, predominantly composed of type I collagen, and by the re-establishment of tissue architecture and mechanical strength.^91^ Key cellular processes include re-epithelialization, collagen fiber cross-linking and alignment, regression of neovasculature, and apoptosis of myofibroblasts once sufficient wound tension is achieved. The interplay between fibroblasts, keratinocytes, endothelial cells, and immune cells determines the structural and functional outcome of the healed tissue.

Emerging evidence suggests that cannabidiol (CBD) exerts modulatory effects during the remodeling phase through its influence on epithelial differentiation, ECM reorganization, and vascular stabilization. In a study by Klein et al.,^81^ topical administration of CBD on traumatic tongue ulcers in Wistar rats resulted in enhanced epithelial tissue structuring and organization during the late stages of healing. Histological evaluation revealed increased epithelial thickness, acanthosis, and hyperkeratinization, accompanied by more organized collagen fiber deposition and pronounced neovascularization. These histopathological features collectively indicate a stimulatory effect of CBD on tissue maturation and ECM remodeling.

Complementary findings from noncutaneous model further support CBD’s role in tissue remodeling. For instance, Zhang et al.^21^ demonstrated that CBD treatment attenuated tissue swelling, fiber rupture, and inflammatory cell infiltration in a murine model of myocardial injury (C57BL/6 mice). These improvements were associated with reduced oxidative stress markers and enhanced organization of myocardial fibers, suggesting a broader role of CBD in postinjury tissue restoration and ECM homeostasis.

Mechanistically, CBD’s regulatory influence on remodeling is thought to involve several convergent pathways. Through activation of cannabinoid receptor type 2 (CB2) and peroxisome proliferator-activated receptor γ (PPARγ), CBD modulates fibroblast activity, promotes collagen maturation, and attenuates excessive matrix metalloproteinase (MMP) activity, thereby preventing aberrant degradation of newly synthesized collagen.^98^ Additionally, CBD’s antioxidant capacity mitigates reactive oxygen species (ROS)-induced collagen cross-link disruption, while its anti-inflammatory effects contribute to the resolution of the wound milieu, allowing for balanced fibroblast-to-myofibroblast transition and appropriate ECM turnover.

CBD may also influence keratinocyte behavior during re-epithelialization. Studies have shown that cannabinoids can regulate keratinocyte proliferation and differentiation through TRPV1, CB1, and CB2 receptor-mediated pathways.^99,100^ By modulating intracellular calcium signaling and cytokine release, CBD may foster a microenvironment conducive to epithelial barrier restoration and stratified epidermal regeneration—processes essential for complete tissue closure and long-term wound stability.

Despite these promising indications, current literature remains insufficient to establish a definitive understanding of CBD’s role in ECM remodeling and re-epithelialization. The available studies are limited by heterogeneous experimental designs, variations in CBD concentrations, and lack of standardization regarding formulation type (topical vs systemic) and administration timing. Furthermore, long-term effects on scar quality, tensile strength, and fibrosis risk have not been systematically evaluated.

Future investigations should integrate quantitative biomechanical assessments, molecular profiling of ECM components (e.g., type I/III collagen ratio, MMP/TIMP balance), and high-resolution imaging techniques to clarify CBD’s influence on the architectural and functional outcomes of tissue remodeling. Elucidating the optimal dosage and delivery method will be crucial for translating CBD’s reparative potential into therapeutic strategies for chronic wounds, fibrotic disorders, and implant-tissue interfaces.

## 4 Biological and Chemical Mechanisms of CBD on Bone Cells

Bone healing is a dynamic and tightly regulated process comprising three overlapping phases: inflammation, repair, and remodeling.^101^ Intraoral bone remodeling is also influenced by the mechanical load from mastication.^102^ Unlike extraoral skeletal sites where remodeling is predominantly regulated by systemic factors such as estrogen deficiency, parathyroid hormone levels, aging, and biomechanical stimuli, intraoral bone is persistently challenged by microbial stimuli.^103–105^ Continuous exposure to microbial challenge decisively modulates the cellular and molecular mechanisms involved in tissue repair, characterizing bone healing in the intraoral region as a unique osteoimmune microenvironment.^106^

During the remodeling phase, bone turnover depends on the rigorously coordinated coupling between osteoclast-mediated resorption and osteoblast-driven bone formation.^107^ In intraoral sites, this coupling is frequently compromised by chronic low-grade inflammation triggered by dysbiotic biofilms enriched with key pathogens such as *P. gingivalis*, *Treponema denticola*, and *Tannerella forsythia*.^108,109^ These microorganisms activate host pattern recognition receptors, particularly Toll-like receptors (TLR2 and TLR4), in resident immune cells, osteoblasts, and periodontal ligament fibroblasts, leading to NF-κB activation and the transcription of pro-inflammatory cytokines such as TNF-α, IL-1β, and IL-6.^110^

Another fundamental mechanism for intraoral bone resorption is the dysregulation of the RANK/RANKL/OPG axis.^109^ In situations such as periodontal inflammation, there is an increase in the expression of receptor activator of nuclear factor kappa B (RANKL) ligand by activated T and B lymphocytes, osteoblastic lineage cells, and periodontal ligament cells, while simultaneously reducing the levels of its decoy receptor, osteoprotegerin (OPG).^109^ This increased RANKL/OPG ratio intensifies the binding of RANKL to RANK in osteoclastic precursors, activating intracellular signaling pathways such as TRAF6, NF-κB, MAPKs, and NFATc.^111^ This signaling cascade promotes the differentiation, polarization, and fusion of multinucleated osteoclasts, increasing the expression of tartrate-resistant acid phosphatase (TRAP), cathepsin K, and integrin αvβ3, culminating in extracellular matrix degradation and mineral dissolution. In addition, mechanisms initiated by virulence factors such as lipopolysaccharides (LPS) from Gram-negative anaerobes can directly or indirectly stimulate the differentiation of osteoclastic precursors through the activation of stromal and immune cells.^112^ Furthermore, Th17 cells and IL-17 have been shown to amplify osteoclastogenic signaling, connecting adaptive immunity to alveolar bone destruction.^113^ Collectively, evidence from osteoimmunology demonstrates that intraoral bone remodeling represents a paradigmatic model of inflammation-induced bone loss, in which host-microorganism interactions critically determine the balance between tissue regeneration and destruction.

Previous studies have demonstrated that CBD, the main nonpsychoactive component of cannabis, can enhance healing and recovery after bone injuries.^93,114–116^ The current knowledge is rather limited and partly contradictory regarding the effect of exogenous cannabinoids on bone cells, and the more elaborate knowledge on the endocannabinoid system with respect to bone. Bone wellness depends on the coordinated action of osteoclast (OC) and osteoblast (OB) cells. The OC is a multinucleated bone cell derived from the hematopoietic lineage and formed through fusion of mononucleated precursors of the myeloid lineage. While OC cells can resorb bone matrix, OB are the bone-forming cells originating from the mesenchymal lineage. From an inflammatory perspective, OC and OB cells have been shown to express CB1 and CB2 receptors, both involved in the pathways of the endocannabinoid system.^117^ CBD has been demonstrated to function as a molecule that acts like a noncompetitive antagonist for CB1 and CB2, primarily for CB,^118,119^ inhibiting the receptor’s activity without directly blocking the agonist binding site. However, inconsistent information has been reported regarding the effects of endocannabinoids on OCs. In a controlled *in vitro* study, Idris et al.^120^ observed a stimulation of RANKL-induced OC formation by the addition of the substances: HU308, 2-AG or AEA, in the cell culture medium. Conversely, in 2016, Ofek et al.^121^ observed an opposite effect, i.e., the inhibition of RANKL-induced OC formation by HU308, as a selective CB2 endocannabinoid agonist.^121^ In fact, AEA reveals a conflicting role, since this endocannabinoid agonist may stimulate human OCs to form actin rings as well as to resorb bone *in vitro*, depending on its concentration. More recently, Nielsen et al.^122^ raised an important question regarding the need to ensure that the dosage of CBD used to treat cultured cells is of pharmacological relevance. In this sense, the authors found that no or only weak inhibition of OC differentiation occurred in the lower doses tested (≤10 μM). Indeed, the number of nuclei per osteoclast cell significantly impacts its function, particularly in bone resorption. Previous studies have already indicated a strong correlation between number of nuclei and OC morphology, with bone formation/resorption, strongly suggesting that multinucleated osteoclast improves resorption efficiency^123^ (Figure 6).

**Figure 6:**
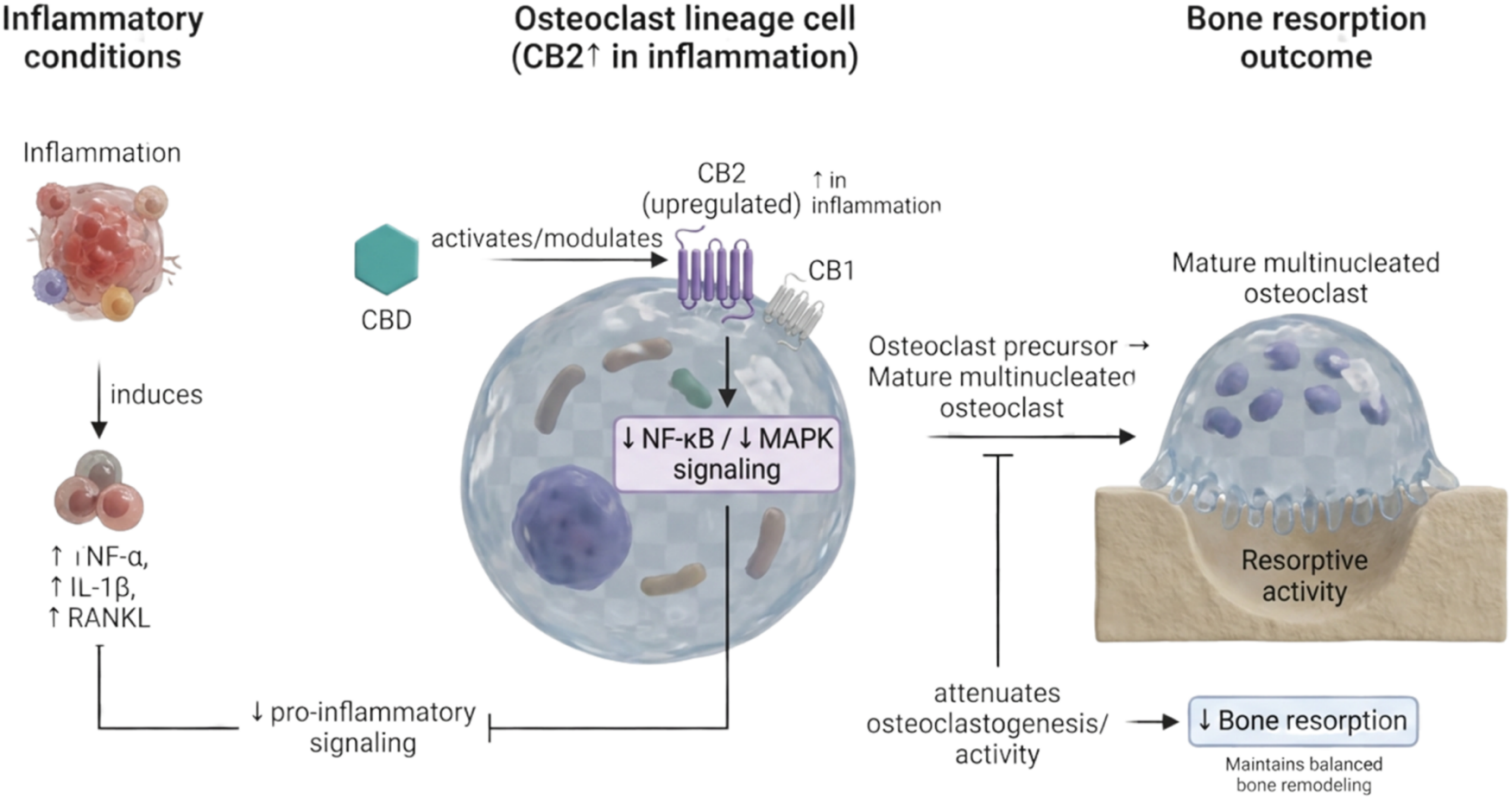
Proposed mechanisms underlying the modulatory effects of cannabidiol (CBD) on bone resorption. Cannabidiol may influence bone remodeling by modulating osteoclast (OC) differentiation and activity through interaction with the endocannabinoid system. Both cannabinoid receptors CB1 and CB2 are expressed in osteoclast lineage cells, and CB2 expression is markedly upregulated under inflammatory conditions, suggesting an active role in the regulation of osteoclastogenesis and bone turnover. Multinucleated, mature osteoclasts exhibit enhanced resorptive capacity; however, CBD is proposed to attenuate excessive osteoclastic activity by reducing pro-inflammatory signaling cascades and cytokine production, including tumor necrosis factor-α (TNF-α), interleukin-1β (IL-1β), and receptor activator of nuclear factor κB ligand (RANKL). Through CB2 receptor activation and downstream signaling modulation, CBD may exert anti-inflammatory and homeostatic effects, dampening nuclear factor κB (NF-κB) and mitogen-activated protein kinase (MAPK) pathways while promoting osteoprotective mediators. Collectively, these actions may contribute to a reduction in inflammation-driven bone resorption and to the maintenance of balanced bone remodeling. Created in BioRender. de Avila, E. (2026) https://BioRender.com/11yguh7. *License: IA29EDLMOS*.

Importantly, recent mechanistic study supports that CBD can directly promote osteogenesis under inflammatory conditions in a receptor- and pathway-dependent manner. Li et al. demonstrated that CBD enhanced osteogenic differentiation of bone marrow mesenchymal stem cells (BMSCs) exposed to inflammatory stimulation, restoring osteogenic markers and mineralization while reducing inflammatory mediator expression; critically, these effects were linked to CB2-dependent activation of p38 MAPK signaling, reinforcing the concept that CBD may “re-couple” osteogenesis during inflammation rather than acting solely as an antiresorptive agent.^124^ This is consistent with evidence that CBD can activate or modulate MAPK-related programs associated with osteogenic differentiation, including increased ALP activity, upregulation of osteoblast lineage markers, and enhanced mineral deposition in osteoblast-like cells.^115^

Extending these observations to oral craniofacial contexts, CBD has also been shown to promote odonto/osteogenic responses in dental-derived progenitors. Qi et al. reported that CBD induced odonto/osteogenesis in human dental pulp cells (HDPCs), supporting the feasibility of CBD-mediated hard-tissue regenerative programs in oral settings.^93^ In an inflammatory mimic, Yu et al. further demonstrated that CBD rescued TNF-α–inhibited proliferation, migration, and osteogenic/odontogenic differentiation of dental pulp stem cells (DPSCs), while concomitantly reducing TNF-α–induced expression of pro-inflammatory cytokines (TNF-α, IL-1β, IL-6) and upregulating pro-angiogenic VEGF expression—features highly relevant to bone regeneration and vascularized repair in infected or inflamed microenvironments.^125^ Collectively, these studies strengthen the biological plausibility of CBD as a dual-action modulator in oral bone regeneration, simultaneously counteracting inflammatory suppression of osteogenesis and supporting pro-reparative cellular behaviors (migration, angiogenic signaling) in MSC-like populations.

On the other hand, CBD also influences bone biology through nonclassical targets. Previously, CBD has been reported to modulate the orphan G protein-coupled receptor GPR55, which plays a functional role in bone remodeling.^126^ Whyte et al.^127^ showed that activation of GPR55 by its agonists increased OC polarization and intensified resorptive activity, whereas these effects were markedly reduced in OC derived from GPR55–/– mice and after CBD treatment, consistent with CBD acting as a functional antagonist of GPR55. In addition, CBD has been reported to promote OB migration and mesenchymal stem cell differentiation in vitro via antagonism of GPR55.^128^ More recent evidence reinforces the relevance of the LPI–GPR55 axis as a regulator of osteoclast differentiation and function and provides additional mechanistic support for CBD-mediated antagonism in this pathway. Mosca et al. (2021) demonstrated that GPR55 signaling modulates osteoclast activity in RANKL-driven differentiation systems, where lysophosphatidylinositol (LPI) enhanced osteoclast activity and GPR55 antagonism reduced resorptive function.^129^

Indeed, the stimulatory and inhibitory effects of cannabinoids on bone cells is a complex matter. Although cannabis is portrayed as a dangerous drug, also due to its ability to reduce bone mineral density, CBD, as a nonpsychoactive component of *C. sativa*, also presents therapeutic properties in bone repair/remodeling that are not yet fully understood. In 2020, Kang et al.^115^ investigated the impact of CBD on osteoblastic differentiation and found that the CBD treatment upregulated the expression of angiopoietin-1, enhanced alkaline phosphatase (ALP) activity, and stimulated cell migration and calcium deposition. The results also demonstrated a time-dependent increase in the expression of osteoblast-related proteins that induce bone, including distal-less homeobox 5 (DLX5), bone sialoprotein (BSP), osteocalcin (OCN), type I collagen, Runx2, osterix (OSX), and ALP matrix-related mineralization.^115^

Mechanistically, Kang et al. (2020) further linked these osteogenic effects to strengthened interactions among RUNX2/OSX and phosphorylated p38 MAPK, suggesting that CBD can facilitate transcriptional control of osteoblast lineage commitment via MAPK-driven osteogenic signaling.^115^ Together with the CB2–p38-dependent osteogenic rescue observed in inflammatory BMSC models,^130^ these findings support a convergent theme in which CBD may promote osteoblastogenesis through p38 MAPK–associated pathways, with receptor dependence likely varying by cell type and inflammatory context.

During the repair phase, vascular invasion and collagen matrix deposition lead to callus formation. Fibroblasts contribute by forming a stroma conducive to vascular growth.^102^ In this sense, Kogan et al.^116^ examined the influence of CBD on fracture healing in a rat model and demonstrated that CBD enhanced the biomechanical strength of the fracture callus without affecting its volume or mineral content. The increased mechanical integrity was attributed to upregulation of PLOD1, an enzyme responsible for collagen cross-linking, in osteoblast cultures treated with CBD. These findings highlight the specific role of CBD in improving fracture healing in long bones, increasing the biomechanical quality of the newly formed bone.^116^

This observation is particularly relevant for craniofacial and peri-implant regeneration, where collagen cross-linking and matrix maturation influence the mechanical quality of newly formed bone and the stability of the implant–bone interface. Although long-bone fracture models cannot be directly extrapolated to intraoral bone, the mechanistic emphasis on collagen cross-linking enzymes suggests that CBD may affect not only osteogenic differentiation but also the material properties of repair tissue, an aspect often overlooked in oral regenerative discussions.

The final stage of fracture healing is the remodeling phase, during which bone regains its original architecture, function, and mechanical strength. This process, which can span several months to years, is modulated by mechanical stimuli.^131^ Under axial loading, bone is formed in regions experiencing stress and resorbed in areas where it is not required.^123^ A recent study by Kamali et al.^114^ reported that CBD accelerated healing and improved biomechanical properties in a rat model of critical-sized bone defect through the mesenchymal stem cell migration and osteogenic differentiation, leading to a more effective bone bridge formation at the defect site.^114^ Although literature regarding the effect of CBD on this process remains scarce, the found information reinforces the idea that CBD could be a promising therapeutic option for promoting bone healing and remodeling.

Notably, emerging oral-focused literature highlights an additional translational layer: beyond host cell modulation, CBD may also influence the microbial drivers of osteoimmune dysfunction. For instance, CBD demonstrated antimicrobial activity against multispecies subgingival biofilms *in vitro*, supporting the possibility that CBD-based local delivery platforms might provide dual benefits: reducing microbial burden while modulating host inflammation and bone remodeling pathways.^132^ This is particularly aligned with periodontal and peri-implant pathogenesis, where persistent biofilm challenge sustains NF-κB signaling, elevates RANKL, and prolongs osteoclastogenic cues.

## 5 Mind the Gap: From Laboratorial Studies to Clinical Application

Regarding the commercial availability of cannabinoid-based medicines, only a few have been rigorously tested to assess their safety and efficacy. They have therefore been approved for use at the national level by regulatory agencies. Epidiolex is an oral solution of 98% pure cannabidiol (CBD). The medicine is approved for the treatment of seizures in pediatric patients with Lennox-Gastaut syndrome or Dravet syndrome.^14^ The biological effects of cannabinoids, the major constituents of the ancient medicinal plant *C. sativa* (marijuana) are mediated by two members of the G-protein coupled receptor family, cannabinoid receptors 1 (CB1R) and 2. The CB1R is the prominent subtype in the central nervous system (CNS) and has drawn great attention as a potential therapeutic avenue in several pathological conditions, including neuropsychological disorders and neurodegenerative diseases. Furthermore, cannabinoids also modulate signal transduction pathways and exert profound effects at peripheral sites. Despite the therapeutic potential of cannabinoids, their clinical application has been significantly hindered by their psychoactive effects. In this review, we briefly summarized our knowledge of cannabinoids and the endocannabinoid system, focusing on the CB1R and the CNS, with emphasis on recent breakthroughs in the field. We aim to define several potential roles of cannabinoid receptors in the modulation of signaling pathways and in association with several pathophysiological conditions. We believe that the therapeutic significance of cannabinoids is masked by the adverse effects and here alternative strategies are discussed to take therapeutic advantage of cannabinoids.^14^ Sativex is also a mouth spray formulated from the extract of the *C. sativa* L. plant, containing mainly Δ^9^-THC and CBD in almost equal proportions. It is indicated for the treatment of spasticity.^133^ However, although there are CBD-based medicines, none of them are indicated for promoting tissue and bone regeneration.^134^

Now, CBD is being studied in several preclinical studies, showing surprising results. However, CBD has limitations due to its highly lipophilic nature with a log *P* of 6.3, which represents the logarithm of the partition coefficient of a drug between n-octanol and water and, it has low water solubility, measuring at 12.6 mg/L. These characteristics allow CBD as a Class II substance in the Biopharmaceutical Classification System (BCS), characterizing it as a substance with low water solubility.^135^ CBD is extensively metabolized in the liver, mainly through hydroxylation, forming 7-OH–CBD. This compound undergoes further metabolization, generating various metabolites, which are eliminated from the body via feces and urine.^136^

Bioavailability refers to the proportion of a drug that reaches systemic circulation unchanged. Intravenous administration provides 100% bioavailability, while oral, inhaled, or transdermal routes reduce it due to incomplete absorption. CBD’s bioavailability clearly varies depending on the method of administration.^137^ When clinically applied, the bioavailability of aerosolized CBD has been reported to be capable of generating rapid peaks in plasma concentration between five and 10 min, with significantly higher bioavailability (around 31%) compared to oral administration.^138^ Oral bioavailability was estimated at 6%, mainly due to extensive first-pass metabolism, where drug molecules are metabolized before entering systemic circulation, which can reduce their bioavailability.^139^ Thus, administering cannabinoids via inhalation or oral mucosal routes provides an alternative method that by passes or minimizes extensive first-pass metabolism, as seen with oral cannabinoid administration.^140^

The effective dose of CBD shows great variability between individuals, influenced by factors such as metabolism, body weight, age, gender, and clinical conditions, which represents a challenge for the development of universal release systems.^141^ One study showed that in humans, after ingesting an oral capsule containing 5.4 mg of CBD, the average maximum plasma concentration (Cmax) was 0.93 ng/mL, which was higher in women than in men.^116^ Furthermore, in one experiment, coadministration of lipids with oral CBD increased systemic availability by almost three times in rats.^142^ These findings reinforce the need for personalized strategies to optimize its administration in different patient profiles.

In the past decade, there has been a growing number of studies focused on enhancing the solubility of class II drugs. Schedule II drugs, according to the Biopharmaceutical Classification, substances or chemicals are defined as drugs with a high potential for abuse, with use potentially leading to severe psychological or physical dependence.^143^ The bioavailability of these drugs is directly influenced by their dissolution rate, which is closely related to solubility, consequently, an improved solubility leads to enhanced bioavailability. Various strategies can be employed to optimize the dissolution rate, such as nanonization, which involves reducing the particle size of the active pharmaceutical ingredient (API) to a nanometric scale.^143^ According to the Noyes-Whitney equation, which indicates the dissolution rate of a solid in a solvent, reducing the particle size of a drug increases its surface area, resulting in a proportional increase in the dissolution rate. Consequently, this leads to better absorption of drugs with low solubility.^144^ In this context, the use of nanotechnology as a drug delivery system has gained prominence due to its unique properties, which include advantages in controlling the physicochemical behavior of the drug, such as solubility and release, as well as directing the drug to the target site, reducing adverse effects.^145^ Nanosystems play a vital role in safeguarding drugs from damage in the gastrointestinal region, thereby facilitating the efficient administration of class II drugs to their intended targets.^146^ One notable characteristic of these nanodrug delivery systems is their remarkable versatility in terms of application routes. These routes include parenteral, oral, nasal, pulmonary, ocular, and transdermal routes.^146^ Among various nanomaterials, liposomes, vesicles, micelles, nanoparticles, nanosuspensions, microemulsions, and nanoemulsions are notable^147^ to enhance the solubility of CBD.

Tran et al. developed a CBD-based nanoemulsion to evaluate its regenerative potential and bioavailability using an *in vitro* human corneal substitute model. The formulation demonstrated superior physicochemical stability, retaining 93.57% of cannabidiol (CBD) content, whereas pure CBD exhibited only 53.58% retention after 4 h of exposure.^79^ This difference was attributed to degradation and metabolic susceptibility of the free compound, while the nanoemulsion matrix provided a protective environment that enhanced CBD chemical stability during topical application.^79^

In the context of dental applications, recent investigations have explored CBD-loaded biomaterials as local delivery platforms, particularly in scenarios requiring prolonged retention and controlled release within the oral cavity. For example, a chitosan-based mucoadhesive hydrogel incorporating CBD-loaded poly­(lactic-*co*-glycolic acid) (PLGA) nanospheres was designed to improve mucosal permeability and local drug availability. In vitro analyses demonstrated a pro-reparative profile characterized by downregulation of inflammatory cytokines, suggesting potential therapeutic applicability in oral inflammatory conditions such as gingivitis and periodontitis.^148^ Furthermore, CBD has been incorporated into multifunctional regenerative hydrogels targeting bone repair. An alginate-based copper-CBD hydrogel exhibiting antibacterial properties was shown to enhance both osteogenic differentiation and angiogenic responses in vitro.^149^ Collectively, these biomaterial systems highlight the potential of nanotechnology-enabled approaches to optimize local CBD delivery to oral tissues; however, robust clinical validation remains lacking.

To date, relatively few studies have investigated nanotechnology-based strategies to enhance the biological performance of CBD, and the available literature is limited by the scarcity of comprehensive preclinical and clinical investigations. Therefore, further well-designed in vitro and in vivo studies are warranted to elucidate the therapeutic benefits of CBD and to clarify its underlying mechanisms of action in wound healing and bone regeneration, thereby facilitating translation into clinical practice.

### 5.1 Legal Status of Cannabidiol

In November 2017, the World Health Organization (WHO)^150^ Expert Committee on Drug Dependence (ECDD) concluded that pure CBD does not appear to pose a risk of abuse or cause harm. The current evidence does not justify reclassifying this substance. However, the decision on its legal status is the responsibility of the legislators in each country. Some countries, such as Australia, Canada, Switzerland, the United Kingdom, and the United States, have loosened regulations on CBD, classifying CBD-containing products as medicines.

Furthermore, in 2018, the Food and Drug Administration (FDA) approved the use of Epidiolex, a CBD-based drug, for the treatment of seizures associated with tuberous sclerosis complex in patients aged one year and older after concluding that the drug is safe and effective for this indication. Following its approval, Epidiolex was classified as a Schedule V substance, the least restrictive category, reserved for medicines with a low potential for abuse. This removed it from the Schedule I classification, which includes substances with a high potential for abuse and no accepted medicinal value under the Federal Controlled Substances Act of 1970.

In 2019, the European Medicines Agency (EMA) approved the first cannabinoid-derived medicine. This drug, composed of isolated CBD, has been authorized for the treatment of children with intractable epilepsy and has been designated an orphan drug because there are no similar therapeutic alternatives available.

Also, in 2019, the National Health Surveillance Agency (Anvisa)^151^ in Brazil regulated the manufacture and sale of CBD-based products in pharmacies on prescription to treat serious conditions such as refractory epilepsy, autism, and degenerative neurological diseases. The agency has imposed limits on the THC content: products with up to 0.2% can be prescribed for a wide range of patients, while those with a higher content are restricted to patients in palliative care or with no other therapeutic options.

On the other hand, in most Latin American countries, the medicinal use of cannabidiol (CBD) is authorized, especially for the treatment of specific diseases such as epilepsy and other neurodegenerative conditions. However, regulations regarding the production, sale, and import of CBD-based products increase considerably between countries. In addition, the recreational use of cannabis remains illegal in almost the entire region, of one except Uruguay. The trend is toward a gradual relaxation of the laws, accompanied by the implementation of standards that ensure the safe and regulated use of the substance.^152^

Despite the advances in legislation, there is still a need to carry out more research, establish clear and accessible regulations, and maintain continuous monitoring to guarantee the efficacy and safety of CBD use as a therapeutic medicine.

## 6 Conclusion

In conclusion, this critical review examined the biological mechanisms through which cannabidiol (CBD) may contribute to wound healing and bone repair/regeneration. Current preclinical evidence supports the therapeutic potential of CBD in enhancing both soft- and hard-tissue repair by modulating key molecular pathways involved in inflammation, cellular proliferation, angiogenesis, and extracellular matrix remodeling. Despite these promising findings, important translational challenges remain. In particular, the minimum effective dose required to achieve therapeutic benefits while minimizing adverse effects has not yet been clearly established, underscoring the need for rigorous dose–response and safety studies to enable clinical application in humans.

With respect to bone repair and regeneration, several mechanistic aspects require further clarification, including the interaction of CBD with osteoblast and osteoclast receptor systems, its effects on osteoclast multinucleation and activity, and the resulting impact on the balance between bone formation and resorption. The studies discussed in this review collectively highlight substantial knowledge gaps regarding both the local and systemic effects of CBD, as well as its integration within complex intracellular signaling networks. Therefore, additional well-designed mechanistic investigations, followed by robust preclinical and clinical studies, are necessary before routine clinical implementation can be considered. Concurrently, comprehensive evaluation of optimal dosage regimens, pharmacokinetics, bioavailability, and bio efficacy of CBD-based drugs and biomaterials will be essential to support their safe and effective translation into oral and craniofacial regenerative therapies.
